# The diversity of *Anopheles *blood feeding patterns suggests different malaria protection strategies in different localities

**DOI:** 10.12688/f1000research.19341.4

**Published:** 2020-10-19

**Authors:** Irfanul Chakim, Tepanata Pumpaibool

**Affiliations:** 1 College of Public Health Sciences, Chulalongkorn University, Bangkok, 10330, Thailand; 2 Faculty of Public Health, Universitas Muhammadiyah Semarang, Kota Semarang, Jawa Tengah, 50273, Indonesia

**Keywords:** Malaria, Anopheles, diversity, blood feeding pattern, protective strategy

## Abstract

**Background:** Malaria is a significant health burden for many countries worldwide. Insecticide-treated bed nets and mosquito repellent are considered effective methods for preventing *Anopheles* bites. However, changes in the biological properties of the vector have led to a reduction in their effectiveness. The vector has been studied, but the behaviour has been poorly examined. Therefore, this study aims to investigate the importance of primary vector activity for selecting an appropriate malaria protection strategy.

**Methods:** Initially, active case detection (ACD) was carried out in western and eastern parts of Indonesia, Jambi and Sumba, to confirm their endemicity level. According to the 2016 national health report of Indonesia, Jambi has an annual parasite index (API) of 0.14 and Sumba has an API of 5.41.

A series of entomological observations were carried out to compare the biting activity of *Anopheles* vectors in two localities, with a total of 216 houses and 216 catchers (108 at each study site).

**Results:** The results indicated that endemicity at the sub-district level is higher than that at the provincial level. Only *Anopheles balabacensi* was found to be exophagic. Multiple comparisons found different biting times between the sites, suggesting that early evening (18.00-20.00) is most likely to be the time when mosquitoes transmit the *Plasmodium* parasite in Jambi, while during sleeping hours (21.00-01.00) is the peak biting time of *Anopheles* mosquitoes in Sumba.

**Conclusions:** The study demonstrates the importance of *Anopheles* species blood feeding patterns in selecting an appropriate malaria protection strategy.

## Introduction

Malaria is a disease that is transmitted by female *Anopheles* vectors. Generally, malaria control is achieved by mass deployment of insecticide-treated bed nets (ITNs) and indoor residual spraying (IRS) and thus such strategy has affected the biological activity of the vector. It has been shown that the distribution of ITNs is responsible for a reduction of 68% in malaria burden in sub-Saharan Africa ^1^. This control method has been widely distributed and a dramatic increase in use has resulted in the mass utilization of ITNs in many countries ^2, 3^. Additionally, personal protection (i.e. repellents) has been found to be effective against mosquito bites and its use has led to a reduction in malaria infection ^4– 6^. However, frequent daily application is required in order to ensure its effectiveness ^7– 9^.

The efficacy of such protection strategies may be problematic as mosquito behavioral activities differ significantly between locations, as observed in Africa, where the vectors exhibit behavioral plasticity ^10– 15^. The shifting behavior of the *Anopheles* vector is a factor that contributes to reduced ITN effectiveness. The behavioral changes of *Anopheles* mosquitoes are in the form of shifts to exophagic behavior ^14, 15^ and biting time modification ^16^. Several findings indicate the ineffectiveness of repellent against malaria infection. The limitations of repellent seem to be related to daily adherence and compliance ^17, 18^ and disproportional utilization ^19^. This issue may be due to the assessment of mosquito protection agents mostly focused on human factor rather than the impact that such types of protection have on the biological property of the vector.

The most effective method against *Anopheles* biting varies between sites and is dependent on the biting activity of the vector. In Uganda, intensive use of ITNs has been suggested due to the biting pattern of *Anopheles gambiae*, with biting mostly occurring late at night, during the time the human population is asleep ^20^. In contrast, bed nets may not provide proper protection against the same *Anopheles* species in Burkina Faso due to an early evening biting time ^21^. Limited studies have investigated *Anopheles* biting patterns in the Indonesian archipelago ^22^. Thus, our study aimed to specifically address the information gap of *Anopheles* biological properties in Indonesia.

## Methods

### Study sites

The sampling was carried out in two localities representing different endemicity areas, namely Jambi province and Sumba Island (Nusa Tenggara Timur Province). Jambi is in Sumatra Island, the western part of Indonesia, geographically situated at 0.45 ° North Latitude, 2.45 ° South Latitude and between 101.10 ° -104.55 ° East Longitude. Sumba Island is situated in the eastern part of Indonesia, with an area of 10,710 km ^2^ and coordinates of 9°40′S 120°00′E. Jambi and Sumba have a total population of 3,515,017 and 685,186, respectively. Jambi has 11 districts with 136 sub-districts and Sumba has four districts with 44 sub-districts. From all of the sub-districts over the sites, the sub-district of each area with the highest number of cases of malaria was selected for our study to be carried out in (Lembah Masurai in Jambi and Kodi Balghar in Sumba). According to the 2016 national health report of Indonesia ^23^, Jambi has an annual parasite index (API) of 0.14 and Sumba has an API of 5.41 ( Table 1).

**Table 1.** Annual parasite index at sub-district and provincial levels.

Area	Number of malaria cases	Collection time (months)	Population ^25, 26^	Yearly incidence rate (sub-district level)	Reported incidence rate (provincial level) [Reference]
Jambi	71	9	26,579	3.56	0.14 [ 23]
Sumba	140	4	21,049	15.96	5.41 [ 23]

### Parasitological investigation

To investigate the API in each sub-district, a series of parasitological assessments were carried out. This assessment was conducted from November 2017 to July 2018 in Jambi and from May to August 2018 in Sumba. Active case detection (ACD) was carried out daily in each site, performed by a local primary healthcare worker. Only people with a tympanic temperature of more than 37.5°C were included in the study. People were asked to go to the local village office for where the finger prick blood sample was collected. Cases were confirmed by light microscopy and prick blood samples were collected directly onto glass slides. A total of 559 and 500 blood samples were taken from Jambi and Sumba, respectively. Two certified independent microscopists assessed all the slides taken from ACD and determined the parasite species.

The API of both sites was calculated using the following formula ^24^:

$$Annual\;parasite\;index=\frac{(\mathrm{total}\;\mathrm{cases}/\mathrm{total}\;\mathrm{months}\;\mathrm{of}\;\mathrm{collection})*12}{\mathrm{total}\;\mathrm{population}\;\mathrm{in}\;\mathrm{each}\;\mathrm{subdistrict}}$$

### Entomological observation

A series of entomological observations were conducted for comparison of the pattern of blood feeding of the potential vector between the two localities. A 24-day observation was done in each area. The human landing catch (HLC) method was used for obtaining *Anopheles* vectors ^22^. The HLC method is a standard method for measuring the exposure of humans to mosquito bites as it directly captures mosquitoes that land and attempt to feed on collectors ^27^. HLC requires an indoor and outdoor catcher present over 12 hours, from 6 pm to 6 am, to reflect the pattern of *Anopheles* biting and blood feeding time preference. The catchers were the owners of the houses and were trained on how to conduct the HLC method. In the current study, indoor and outdoor mosquito collection was carried out at each house ^22^. Six houses were selected for daily HLC and there were six days of collection per a week. Inclusion criteria of the houses was as follows: (1) three houses had to have had a malaria infection during the previous one-year period; (2) the other three houses had to have had an absence of malaria infection for at least one-year and had to be in close proximity to the infected houses. The information about malaria infections at each house was obtained by interviewing each house member. In total, there were 216 houses and 216 catchers (108 at each study site). The observation was carried out 24 days in each study site. Random selection was done for repetition (for example, a house which had indoor collection in the first week would have outdoor collection in the next week and change to indoor in the last week and vice versa); thus, each house had the same pattern of an indoor and outdoor collection. The distance between each house was less than two kilometers to avoid biases due to potential differences in mosquito species abundance. All the mosquito species were confirmed by entomological experts from Eijkman Institute for Molecular Biology, Jakarta, Indonesia by dissection and viewing under a light microscope using the Anopheles identification key developed by *Rattanarithikul et al.* ^28^.

### Statistical analysis

To analyze the data, descriptive and analytical tests were carried out to analyze the mosquito blood feeding pattern of each site. The analysis provided three types of results: 1) the preferred biting time of *Anopheles* mosquitoes at each site by comparing the number of collected mosquitoes in each site using a student t-test statistical method; 2) a comparison of the number of mosquitoes collected indoors and outdoors from each location using the Mann Whitney test; and 3) multiple comparisons of biting time by pooled analysis for each location using the Kruskal-Wallis test and Dunn’s multiple comparison test. All the analyses and comparisons were carried out using GraphPad Software version 8.00 (La Jolla California, USA). Relative abundance and human landing rate (HLR) were calculated using the following formulas:

$$Relative\;abundance=\frac{\text{(}\mathrm{Total}\;\mathrm{mosquitoes}\;\mathrm{collected}\;\mathrm{of}\;\mathrm{each}\;\mathrm{species}*\;100\text{)}}{\mathrm{Total}\;\mathrm{mosquito}\;\mathrm{collection}}$$

$$Human\;landing\;rate=\frac{\mathrm{Total}\;\mathrm{mosquitoes}\;\mathrm{collected}\;\mathrm{of}\;\mathrm{each}\;\mathrm{species}}{\mathrm{Total}\;\mathrm{number}\;\mathrm{of}\;\mathrm{catchers}}$$

### Ethical statement

Informed consent was obtained from collectors performing HLC. Permission was also received from the owner of the house and the community on both sites. Community permission has been obtained by collectively gathering village residents along with the head of the village in the village office. Written informed consent was also sought for every participant of the parasitological assessment. This study was approved by the ethics commission of Universitas Muhammadiyah Semarang [22/EC/FKM/2017].

## Results

The parasitological assessment found a total of 211 cases of malaria in both localities ^29^. Only *Plasmodium vivax* was found in Jambi, responsible for 71 malaria cases. Participants from Jambi were 60.6% male (43) and 39.4% female (28) with a mean age of 15.5 years, ranging from one to 59 years. In Sumba, three types of *Plasmodium* were successfully detected during ACD. From a total of 140 malaria cases in Sumba, 92 (65.7%) were *Plasmodium falciparum*, 43 (30.7%) were *Plasmodium vivax*, and 5 (3.6%) were *Plasmodium malariae*. Participants from Sumba were 58.6% male (82) and 41.4% female (58) with a mean age of 10.9 years, ranging from one to 53 years. The calculated APIs of the two study sites were 3.56 and 15.96, respectively ( Table 1). The API result of this study is different to the national health report of the Ministry of Health, Indonesia. The API is up to 2.95-25.4-fold higher at the sub-district level, found in this report, than at the provincial level, as stated in the report.

A total of 2,435 *Anopheles* mosquitoes were successfully collected from 216 houses and 216 catchers at the two locations (108 houses and catchers at each study site) ^29^. There was a statistical difference in the total number of *Anopheles* mosquitoes caught between Jambi and Sumba (P value= <0.0001, U = 5938). Jambi had mosquito abundance of 71 and Sumba had 2,364. Four *Anopheles* species were successfully collected in Jambi, namely *Anopheles balabacensis*, *Anopheles barbirostris*, *Anopheles maculatus* and *Anopheles sinensis*. *An. balabacensis,* which belongs to Leucosphyrus group, had the highest abundance, as shown with its relative abundance of 78.87 and HLR of 0.52 per person per night, followed by *An. maculatus* (relative abundance: 18.31 and HLR: 0.12 per person per night), *An. barbirostris* (relative abundance: 1.41 and HLR: 0.01 per person per night) and *An. sinensis* (relative abundance: 1.41 and HLR: 0.01 per person per night). In contrast, the dominant *Anopheles* species in Sumba were *Anopheles aconitus* and *Anopheles sundaicus,* with a relative abundance of 40.02 and 58.50 and HLR of 8.76 and 12.81 per person per night, respectively. The other minor species found were *An. barbirostris* (relative abundance: 0.09 and HLR: 0.02), *Anopheles farauti* (relative abundance: 0.04 and HLR: 0.01), *Anopheles leucosphyrus* (relative abundance: 0.04 and HLR: 0.01), *An. maculatus* (relative abundance: 1.06 and HLR: 0.23), *Anopheles subpictus* (relative abundance: 0.17 and HLR: 0.04) and *Anopheles vagus* (relative abundance: 0.09 and HLR: 0.02) ( Table 2).

**Table 2.** Species, total numbers of mosquitoes collected, relative abundance and human landing rate of *Anopheles* mosquitoes from Jambi and Sumba.

Jambi			
Species	Total collection	Relative abundance (%)	Human landing rate
*An. balabacensis*	56	78.87	0.52
*An. barbirostris*	1	1.41	0.01
*An. maculatus*	13	18.31	0.12
*An. sinensis*	1	1.41	0.01
Total	71		0.66
Sumba			
Species	Total collection	Relative abundance (%)	Human landing rate
*An. aconitus*	946	40.02	8.76
*An. barbirostris*	2	0.09	0.02
*An. farauti*	1	0.04	0.01
*An. leucosphyrus*	1	0.04	0.01
*An. maculatus*	25	1.06	0.23
*An. subpictus*	4	0.17	0.04
*An. sundaicus*	1,383	58.50	12.81
*An. vagus*	2	0.09	0.02
Total	2,364		21.90

**Figure 1.**
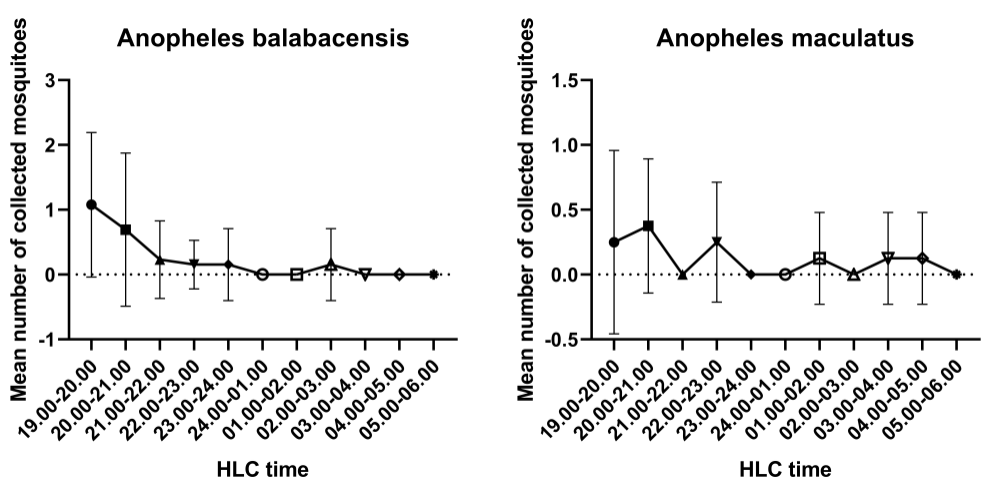
Biting time pattern of *Anopheles balabacensis* and *An. maculatus* collected from Jambi (mean +/- SD). HLC, human landing catch.

**Figure 2.**
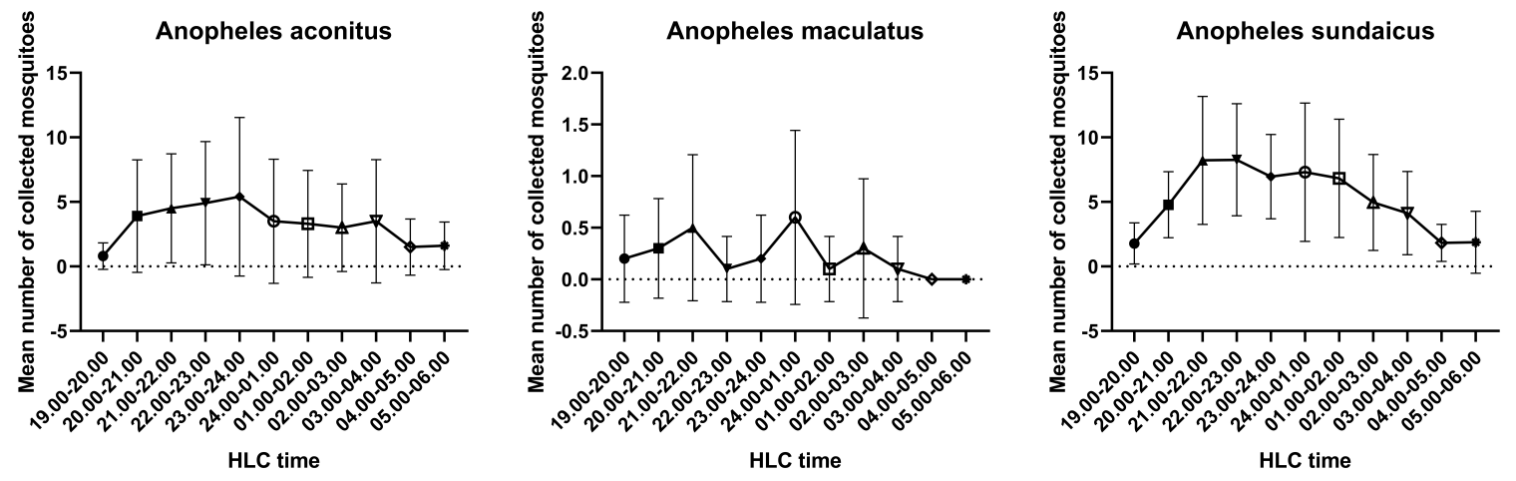
*Anopheles aconitus*, *An. maculatus* and *An. sundaicus* biting times in Sumba (mean +/- SD). HLC, human landing catch.

There was a difference in *Anopheles* biting time between Jambi and Sumba ( Figure 1 and Figure 2). *An. balabacensis* from Jambi has a peak in biting time during early evening (6 pm), which decreases substantially until midnight, while *An. maculatus* showed an irregular biting time pattern. On the other hand, there is a similar trend in biting time between *An. aconistus* and *An. sundaicus* collected from Sumba; it gradually increased until its peak biting time between 21.00-22.00 and 01.00-02.00; then, it decreased progressively until 05.00-06.00. Additionally, an irregular biting time pattern has also been observed for *An. maculatus* from Sumba.

**Figure 3.**
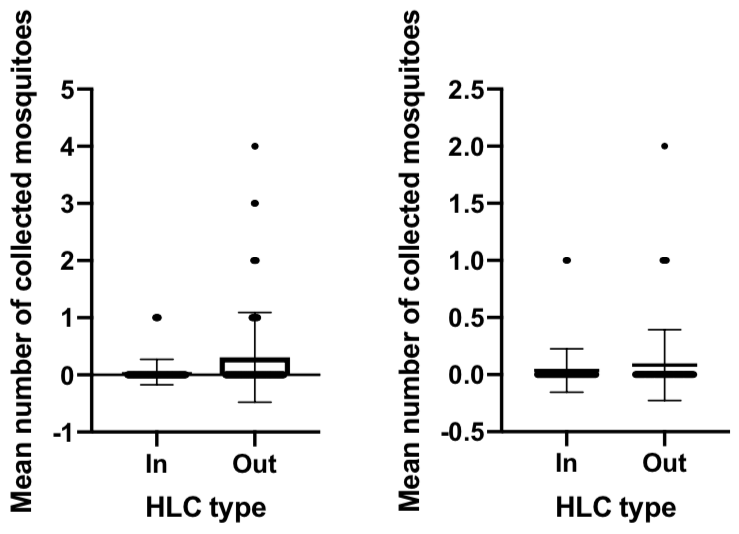
Indoor and outdoor biting preference of *Anopheles balabacensis* (left) and *An. maculatus* (right) in Jambi (mean +/- SD). HLC, human landing catch.

**Figure 4.**
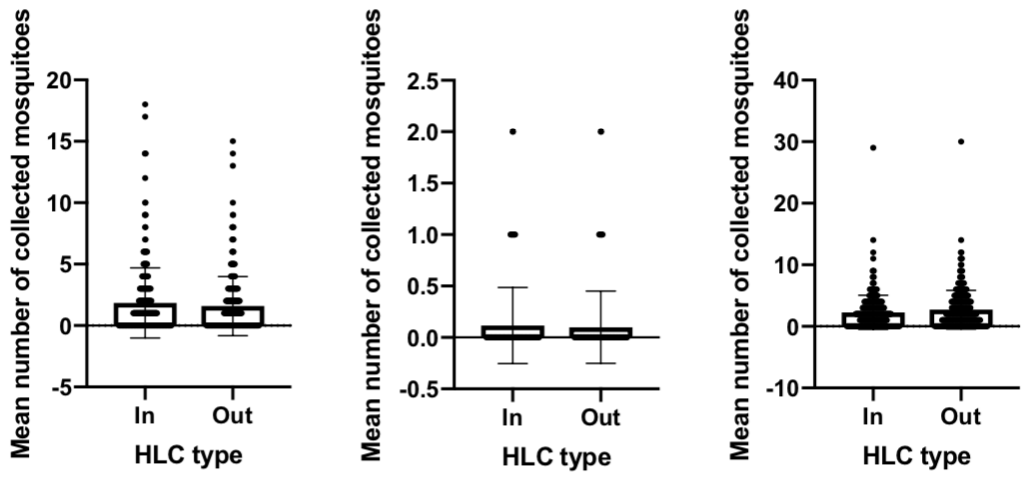
Mean number of *Anopheles aconitus* (left), *An. maculatus* (center) and *An. sundaicus* (right) indoors and outdoors in Sumba (mean +/- SD). HLC, human landing catch.

To investigate the biting preference of *Anopheles* mosquito, an indoor and outdoor comparison was carried out ( Figure 3 and Figure 4). There was a statistically significant finding for the biting preference of *An. balabacensis* from Jambi; the number of collected mosquitoes from outdoor was higher than that of indoor collection (P value = 0.0004, U = 10634). No statistical difference was observed for *An. maculatus* (P value = 0.1163, U = 5614). A similar pattern was found for *An. aconitus* (P value = 0.3481, U = 36423), *An. maculatus* (P value = 0.6623, U = 7202) and *An. sundaicus* (P value = 0.1466, U = 38622), where there was no difference between indoor and outdoor collection, suggesting that undertaking an indoor or outdoor activity carries the same risk of getting mosquito bites.

**Figure 5.**
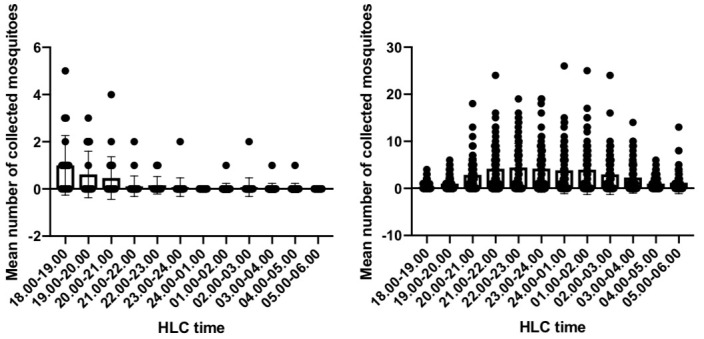
Mean number of *Anopheles* mosquito at different biting times in Jambi (left) and Sumba (right). HLC, human landing catch.

To investigate the difference in mosquito biting times between Jambi and Sumba, a multiple comparison analysis of pooled mosquito sample data was carried out ( Table 3 and Figure 5). Based on the mosquito biting time in Jambi, the number of bites during the early evening (18.00-19.00) was statistically different from other biting times, from 21.00-22.00 to 05.00-06.00 (P value= <0.00001, H = 76.32). Additionally, the number of bites at 19.00-20.00 was statistically different from the number at 24.00-01.00 and 05.00-06.00 (P value= 0.0435, H = 76.32). In Sumba, the number of bites during the early evening at 18.00-19.00 was statistically different from the other biting times, except for 19.00-20.00, 04.00-05.00 and 05.00-06.00 (P value= <0.0001-0.0069). In addition, the number of bites at 19.00-20.00 differed from the number at 21.00-22.00, 22.00-23.00 and 23.00-24.00 (P value= 0.0088-0.0168, H = 9052): 21.00-22.00 differed from 04.00-05.00 and 05.00-06.00 (P value= 0.0030-0.0052, H = 9052); 22.00-23.00 differed from 04.00-05.00 and 05.00-06.00 (P value= 0.0030-0.0050, H = 9052); 23.00-24.00 differed from 04.00-05.00 and 05.00-06.00 (P value= 0.0059-0.0099, H = 9052);and 24.00-01.00 differed from 05.00-06.00 (P value= 0.0347, H = 9052). These results indicate that in Jambi, the peak biting time is during early evening at 18.00-20.00. In Sumba, the mosquitoes started feeding and feeding gradually intensified during the early evening (18.00-21.00), the intensity of the mosquitoes was stable until 02.00 and then the mosquito biting intensity declined during the early morning.

**Table 3.** Summary of significant multiple comparisons between different mosquito biting times in Jambi and Sumba. The test was done using the Kruskal-Wallis test followed by Dunn’s multiple comparison test.

Jambi			
Dunn's multiple comparisons test	Mean rank difference	Summary	Adjusted P value
18.00-19.00 vs. 21.00-22.00	77.81	****	<0.0001
18.00-19.00 vs. 22.00-23.00	67.15	***	0.0004
18.00-19.00 vs. 23.00-24.00	83.48	****	<0.0001
18.00-19.00 vs. 24.00-01.00	89.85	****	<0.0001
18.00-19.00 vs. 01.00-02.00	84.17	****	<0.0001
18.00-19.00 vs. 02.00-03.00	83.48	****	<0.0001
18.00-19.00 vs. 03.00-04.00	84.17	****	<0.0001
18.00-19.00 vs. 04.00-05.00	84.17	****	<0.0001
18.00-19.00 vs. 05.00-06.00	89.85	****	<0.0001
19.00-20.00 vs. 24.00-01.00	50.52	*	0.0435
19.00-20.00 vs. 05.00-06.00	50.52	*	0.0435
Sumba			
Dunn's multiple comparisons test	Mean rank difference	Summary	Adjusted P value
18.00-19.00 vs. 20.00-21.00	-195.5	****	<0.0001
18.00-19.00 vs. 21.00-22.00	-238.1	****	<0.0001
18.00-19.00 vs. 22.00-23.00	-238.3	****	<0.0001
18.00-19.00 vs. 23.00-24.00	-232.0	****	<0.0001
18.00-19.00 vs. 24.00-01.00	-214.9	****	<0.0001
18.00-19.00 vs. 01.00-02.00	-204.8	****	<0.0001
18.00-19.00 vs. 02.00-03.00	-180.1	***	0.0001
18.00-19.00 vs. 03.00-04.00	-147.6	**	0.0069
19.00-20.00 vs. 21.00-22.00	-145.1	**	0.0090
19.00-20.00 vs. 22.00-23.00	-145.4	**	0.0088
19.00-20.00 vs. 23.00-24.00	-139.1	*	0.0168
21.00-22.00 vs. 04.00-05.00	150.2	**	0.0052
21.00-22.00 vs. 05.00-06.00	155.0	**	0.0030
22.00-23.00 vs. 04.00-05.00	150.5	**	0.0050
22.00-23.00 vs. 05.00-06.00	155.2	**	0.0030
23.00-24.00 vs. 04.00-05.00	144.2	**	0.0099
23.00-24.00 vs. 05.00-06.00	149.0	**	0.0059
24.00-01.00 vs. 05.00-06.00	131.9	*	0.0347

* <0.05, ** <0.01, *** <0.001, **** <0.0001.

## Discussion

According to the Malaria Atlas Project ^30^, for API <0.1, *Plasmodium falciparum* and *Plasmodium vivax* distributions are similar across the Indonesian archipelago. *Plasmodium falciparum* is more stable in distribution, where each part of Indonesian archipelago has the same pattern of low to moderate API. Meanwhile, *Plasmodium vivax* is more intense in the eastern part of Indonesia and unstably distributed in the western part of Indonesia. However, only *Plasmodium vivax* was found in Jambi, and more diverse *Plasmodium* species have been observed in Sumba, suggesting a different diversity of *Plasmodium* species distribution in the two localities. A discrepancy was also found in the calculated API between this study and the basic health report by the Ministry of Health of Indonesia, which might be explained by the different ways of presenting the data. The national health report ^23^ used the provincial population and the larger the area, the larger the population involved in the calculation, as API is calculated by dividing the total cases and the total population. API at a sub-district level is often observed to vary from one district to another and variation between districts is observed at a provincial level ^31, 32^.

There are 20 *Anopheles* species known to be vectors for malaria in Indonesia. In this study, four and eight species have been found in Jambi and Sumba, respectively. The student t-test suggested a different abundance in the number of *Anopheles* mosquitoes between the two sites. This difference is often explained by environmental conditions. A distinct sampling time may cause this difference in mosquito abundance; however, since rainfall anomalies have been observed in Indonesia, this may not be the case ^33^. Since the existence of Anopheles breeding sites depends on rainfall providing a sufficient water bodies for the mosquitoes to lay eggs, rainfall anomalies in Indonesia may lead to an irregular pattern of mosquito abundance across time and place in Indonesia. The limited number of water bodies or humidity conditions may affect the habitat and abundance of *Anopheles* mosquitoes in Jambi ^34, 35^. The difference in the annual incidence rate of malaria infection may also reflect mosquito abundance in different endemic areas. However, no correlation may be found if the correlation of annual incidence rate and mosquito abundance takes into account the species of *Plasmodium* ^36^.

The main *Anopheles* vector and biting preference differs between Jambi and Sumba. *An. balabencis,* which belongs to leucosphyrus group, is the primary vector in Jambi, as determined from its highest relative abundance and HLR. Moreover, *An. aconitus* and *An. sundaicus* are the primary vectors in Sumba, along with other minor *Anopheles* species found. Only *An. balabacensis* in Jambi was found to be exophagic, as previously known from the biting preference of this peculiar species ^37^. *An. maculatus* has been found to be both endophagic or exophagic similar to the finding of Elyazar *et al.* ^37^. However, previous studies have found that *An. aconitus* has an irregular pattern of biting preference while *An. sundaicus* is mainly exophagic ^37^. This study found that there was no significant difference between the indoor and outdoor biting preference of *An. aconitus* and *An. sundaicus*, suggesting that these species can be both endophagic and exophagic.

Biting time is essential to understanding the underlying biological properties of mosquitoes and to avoid *Anopheles* bites to control malaria infection. The data obtained suggest different biting times of *Anopheles* in Jambi and Sumba. Early evening (18.00-20.00) is most likely to be the mosquito feeding time in Jambi, when most people are undertaking activities and are unprotected. However, in the late evening (21.00-02.00), more people in Sumba may get *Anopheles* bites, reflecting sleeping time, when Sumbanese people may be vulnerable to infection with malaria parasites. This suggests the importance of ITNs for evading malaria infection in Sumba. The biting time of *Anopheles* in Jambi is similar to that in Halmahera, Maluku Island ^22^. However, the finding from Sumba Island is different from other parts of Indonesia, which shows a gradual increase or decrease in the number of Anopheles mosquitoes in accordance with its biting time ^22^. Furthermore, the difference in mosquito biting activity in each location could be simply explained by its dominant species at each location. For example, the early biting *Anopheles* activity in Jambi is explained by its dominant species of *Anopheles balabacensis* that exhibit an early biting time. Limited studies have tried to describe mosquito biting patterns in relation to the selection of malaria control strategies ^20, 21^. This finding strengthens the previous report that effective malaria prevention depends on local *Anopheles* vector biting behavior. *Anopheles* vectors in Jambi share the same behavior as those in Burkina Faso, where bed net protection may not be effective for preventing biting exposure as *Anopheles* species in the area are dominant in the early evening ^21^. In contrast, similar to Uganda, intensive use of ITNs combined with indoor residual spraying is the most effective protection approach for Sumba Island for avoiding malaria infection ^20^. Interestingly, studies conducted in Solomon island suggested that *Anopheles farauti* has a similar pattern of early night and outdoor biting behavior ^38– 40^. Although, these studies recommended that LLINs and IRS are still significantly effective in reducing transmission based on the feeding cycle of *Anopheles farauti*, which is far shorter than the *Plasmodium falciparum* or *Plasmodium vivax* extrinsic incubation period. However, in an area in which the feeding cycle of the vector is unknown, study will be challenging. Additionally, our study also suggests that a vector control implementation will need to consider the dominant vector species, as a different location may have a different predominant *Anopheles* species, as well as continuous monitoring of such assessment via sentinel sites ^41^.

Biting preference has previously been known to have an underlying genetic background ^42^. For instance, chromosome inversions of *2Rbc*, *2Ra* and *3Ra* are associated with exophagic and endophagic behavior in some *Anopheles* species ^43, 44^. However, genetic background may vary within the genus and among mosquitoes within the same species in different locations ^45^. The finding also suggests that differences in *Anopheles* biting time may be an effect of different genetic backgrounds. Further research might explore this aspect. 

There are some limitations of the current study. There was no intervention included to measure the effectiveness of any type of protection in correlation with the different biting times in each study site. In further research, an intervention approach should be used to find the best protection strategy in locations that may have different Anopheles biting times. Additionally, our collection method was limited to three weeks observational research. A more prolonged study needs to be conducted to reflect yearly fluctuations in local *Anopheles* biting times.

## Conclusion

In conclusion, this study suggests four important findings for public health control: (1) API may be significantly lower at the provincial level compared to the sub-district level and varied accordingly, suggesting that malaria foci may be maintained in a locality from a provincial level, especially in areas of low to moderate endemicity; (2) the importance of mosquito abundance information may reflect malaria incidence rate in a location ^46, 47^; (3) all *Anopheles* species, except *An. balabacnesis*, can be both endophagic and exophagic, suggesting a comprehensive protection approach is required to avoid mosquito bites regardless of being indoors or outdoors; (4) biting time may suggest the use a different prevention approach in each area; for example, people in Jambi may need to use mosquito repellent during activities in the early evening, while ITNs combined with indoor residual spraying may need to be deployed to protect malaria infection during sleeping hours in Sumba.

## Data availability

### Underlying data

Zenodo: The diversity of Anopheles blood feeding patterns suggest different malaria protection strategies in different localities. https://doi.org/10.5281/zenodo.3269824 ^29^

This project contains the following underlying data:

- Supplementary 1.xls (The total number of mosquitoes collected, number collected per time period and number collected indoors/outdoors)
- Supplementary 2.xls (The number of mosquitoes caught for each species in Jambi and relative abundance and HLC calculations)
- Supplementary 3.xls (The number of mosquitoes caught for each species in Sumba and relative abundance and HLC calculations)
- Supplementary 4.xls (Results of all Dunn’s multiple comparisons tests for biting times in Jambi and Sumba)
- Supplementary 5.docx (Flow chart of the HLC collection method)
- Supplementary 6.rar (detailed data of all *Anopheles* found in Jambi per collection type and collection time)
- Supplementary 7.zip (detailed data of all *Anopheles* found in Sumba per collection type and collection time)
- Supplementary 8.xlsx (demographic data and parasite species for participants from both study sites)

Data are available under the terms of the Creative Commons Attribution 4.0 International license (CC-BY 4.0).
