# Supplementary material for: The diversity of Anopheles blood feeding patterns suggests different malaria protection strategies in different localities
Source: F1000Res. 2020 Oct 19;8:1217. Originally published 2019 Jul 30. [Version 4] doi: 10.12688/f1000research.19341.4 (PMC7578570; doi:10.12688/f1000research.19341.4)
Supplement: Peer review history

# Peer review: Reviewer response for version 4

**DOI:** 10.5256/f1000research.29652.r73312

**Published:** 2020-10-20

**Authors:** Shüné V. Oliver

**Affiliations:** 1 Centre for Emerging, Zoonotic and Parasitic Diseases Centre for Emerging, Zoonotic and Parasitic Diseases, National Institute for Communicable Diseases, Johannesburg, South Africa; 2 Wits Research Institute for Malaria, Johannesburg, South Africa

I have no further comments to make on the manuscript.

Is the work clearly and accurately presented and does it cite the current literature?

Yes

If applicable, is the statistical analysis and its interpretation appropriate?

Partly

Are all the source data underlying the results available to ensure full reproducibility?

Yes

Is the study design appropriate and is the work technically sound?

Yes

Are the conclusions drawn adequately supported by the results?

Yes

Are sufficient details of methods and analysis provided to allow replication by others?

Yes

Reviewer Expertise:

Anopheles vector biology

I confirm that I have read this submission and believe that I have an appropriate level of expertise to confirm that it is of an acceptable scientific standard.

# Peer review: Reviewer response for version 3

**DOI:** 10.5256/f1000research.24735.r67103

**Published:** 2020-07-20

**Authors:** Shüné V. Oliver

**Affiliations:** 1 Centre for Emerging, Zoonotic and Parasitic Diseases Centre for Emerging, Zoonotic and Parasitic Diseases, National Institute for Communicable Diseases, Johannesburg, South Africa; 2 Wits Research Institute for Malaria, Johannesburg, South Africa

The authors have made efforts to attend to the comments that have been given before. I have made a few comments on the PDF regarding the small corrections that need to be made - please see the PDF here.

 I have only one small comment that must be addressed and the manuscript cannot be published without these corrections. This was mentioned before, but there are two points regarding the statistics.

 The first is that the there needs to be consistency with the number of significant figures quoted in the p-value. Secondly, the p-value is not the only statistical indicator that must be quoted. It is essential that the degrees of freedom, T-stats, F-stats are quoted. This must be corrected before the MS is published.

Is the work clearly and accurately presented and does it cite the current literature?

Yes

If applicable, is the statistical analysis and its interpretation appropriate?

Partly

Are all the source data underlying the results available to ensure full reproducibility?

Yes

Is the study design appropriate and is the work technically sound?

Yes

Are the conclusions drawn adequately supported by the results?

Yes

Are sufficient details of methods and analysis provided to allow replication by others?

Yes

Reviewer Expertise:

Anopheles vector biology

I confirm that I have read this submission and believe that I have an appropriate level of expertise to confirm that it is of an acceptable scientific standard, however I have significant reservations, as outlined above.

# Peer review: Reviewer response for version 3

**DOI:** 10.5256/f1000research.24735.r60142

**Published:** 2020-03-10

**Authors:** Giles E. Duffield

**Affiliations:** 1 Department of Biological Sciences and Eck Institute for Global Health, Galvin Life Science Center, University of Notre Dame, Notre Dame, USA

The authors have addressed the comments appropriately and the revisions to the manuscript are complete. Congratulations on publishing this interesting study.

Is the work clearly and accurately presented and does it cite the current literature?

Partly

If applicable, is the statistical analysis and its interpretation appropriate?

Partly

Are all the source data underlying the results available to ensure full reproducibility?

Yes

Is the study design appropriate and is the work technically sound?

Yes

Are the conclusions drawn adequately supported by the results?

Yes

Are sufficient details of methods and analysis provided to allow replication by others?

Yes

Reviewer Expertise:

Biological timing of mosquito vectors

I confirm that I have read this submission and believe that I have an appropriate level of expertise to confirm that it is of an acceptable scientific standard.

# Peer review: Reviewer response for version 2

**DOI:** 10.5256/f1000research.23311.r56189

**Published:** 2019-12-05

**Authors:** Giles E. Duffield

**Affiliations:** 1 Department of Biological Sciences and Eck Institute for Global Health, Galvin Life Science Center, University of Notre Dame, Notre Dame, USA

Thank you for clarifying the status of the error bars on the charts in the response to the reviewers comments. However, please state in the figure legends what the data points are showing in each figure so that the reader of the manuscript will be informed. i.e. mean +/- SD if that is the case.

 It is unclear what statistical tests have produced the data shown in Table 3 and in the text shown on page 7. According to the methods section, the authors state that they applied Student’s t-tests, Mann whitney tests, and Kruskall-wallis followed by Dunn’s post hoc tests. Please clarify in the manuscript results section and table/figure legends what tests have been applied specifically rather than simply providing p values. It would be appropriate to apply the Kruskall-wallis followed by Dunn’s post hoc tests in the places where the comparison involve greater than 3 groups per test; and where simple pairwise comparisons are made, it would be appropriate that tests should be multiple means corrected. Note that the data could be cube-root or square-root transformed in an effort to correct for non-normal distributions, thereby facilitating parametric analysis such as by ANOVA.

 The authors stated they had corrected the italicization of 'Anopheles' throughout. This is not the case.

 Paragraph 1 of Introduction: It was surprising to see a paraphrasing of the reviewers text "(i.e. repellents, barriers, clothing, head-nets, etc)." This was just a guess as to what the authors were referring to in terms of "personal protection".

 Last sentence of methods section of abstract has grammatical errors in it. Please refer back to original suggested text change in earlier Reviewers comments.

Is the work clearly and accurately presented and does it cite the current literature?

Partly

If applicable, is the statistical analysis and its interpretation appropriate?

Partly

Are all the source data underlying the results available to ensure full reproducibility?

Yes

Is the study design appropriate and is the work technically sound?

Yes

Are the conclusions drawn adequately supported by the results?

Yes

Are sufficient details of methods and analysis provided to allow replication by others?

Yes

Reviewer Expertise:

Biological timing of mosquito vectors

I confirm that I have read this submission and believe that I have an appropriate level of expertise to confirm that it is of an acceptable scientific standard, however I have significant reservations, as outlined above.

# Peer review: Reviewer response for version 1

**DOI:** 10.5256/f1000research.21201.r54515

**Published:** 2019-10-22

**Authors:** Giles E. Duffield

**Affiliations:** 1 Department of Biological Sciences and Eck Institute for Global Health, Galvin Life Science Center, University of Notre Dame, Notre Dame, USA

This study contributes to a developing insight into the efficacy of malaria control interventions that focus on the mosquito vector and its behaviour. It is fast becoming apparent that the success of interventions such as barrier nets, insecticides and other interventions is highly dependent upon the specific temporal and spatial behavior of the mosquito and its relationship to the patterns of human activity in each locality. Some of this is due to the species composition and in some cases, there is evidence for a shift in the behavior due to previous and/or ongoing selective pressures. The current study provides detailed evidence for distinct differences in the biting behaviours of Anopheles in two different locations in Indonesia. Such differences may be important in explaining differences in rates of malaria transmission, and may be useful in modifying current intervention methods. The manuscript is well written, and easy to follow. It represents a well-executed study and presents interesting data.

**Major Comments: **

1. Table 3 and related results. For the purposes of scientific rigor, I suggest the authors employ multiple means corrected statistics rather than repeated single Students t-tests, such as an ANOVA followed by post hoc multiple means corrected pairwise tests such as Bonferroni, Tukey, Dunnett’s tests.
2. Page 9. Paragraph 2. Discussion. The authors state that “…circadian clock genes are associated with exophagic and endophagic behavior in some Anopheles species. However, these studies do not report such findings. In fact, Malita *et al.* (2016) ^1^ states the opposite, that they do not find any association between biting time or biting location and circadian clock gene polymorphisms.
3. The authors compare their results with studies of African populations of Anopheles. I think much can also be gleaned from relating the current study with the work conducted in the Solomon Islands and PNG. I am listing a few of those that may be relevant for consideration, although this is not an exhaustive list:
  Frequent blood feeding enables insecticide-treated nets to reduce transmission by mosquitoes that bite predominately outdoors. Russell TL, Beebe NW, Bugoro H, Apairamo A, Chow WK, Cooper RD, Collins FH, Lobo NF, Burkot TR. Malar J. 2016 Mar 10;15:156. doi: 10.1186/s12936-016-1195-8. ^2^
  Determinants of host feeding success by Anopheles farauti. Russell TL, Beebe NW, Bugoro H, Apairamo A, Cooper RD, Collins FH, Lobo NF, Burkot TR.Malar J. 2016 Mar 10;15:152. doi: 10.1186/s12936-016-1168-y. ^3^
  Anopheles farauti is a homogeneous population that blood feeds early and outdoors in the Solomon Islands.Russell TL, Beebe NW, Bugoro H, Apairamo A, Collins FH, Cooper RD, Lobo NF, Burkot TR.Malar J. 2016 Mar 9;15:151. doi: 10.1186/s12936-016-1194-9. ^4^
  Successful malaria elimination strategies require interventions that target changing vector behaviours. Russell TL, Beebe NW, Cooper RD, Lobo NF, Burkot TR. Malar J. 2013 Feb 7;12:56. doi: 10.1186/1475-2875-12-56. ^5^
4. More could be discussed by the authors as to the relevance of the specifies specific dominance in each location. For example, much of the temporal biting profile for *Anopheles* mosquitoes in Jambi can presumably be explain simply by the dominant species at that location being *A. balabacensis*, which they authors have demonstrated to exhibit an early evening biting profile (Fig. 1). I think this simple species location effect could be stated more clearly as an obvious explanation for the differences in general ‘all-species’ assessment of anopheline biting activity at each location (Fig. 5). 

**Minor Comments** **:** 

1. Abstract. Methods section. The last sentence is ambiguous. Suggest rewrite as “..vectors in two localities, with a total of 216 houses and 216 catchers (108 at each study site).”
2. Throughout text, “mosquitoes” is incorrectly spelt.
3. Throughout text, there is inconsistent italicized “Anopheles”.
4. Introduction, paragraph 1. Please define better “personal protection”. Presumably this is the use of repellents, barriers, clothing, head-nets, etc.
5. Figure 1. Are these SEM or SD bars on the charts?
6. Discussion: 2 ^nd^ paragraph, “The difference in mosquito abundance may also reflect the annual incidence rate of malaria infection in different endemic areas”. Should this not be argued in the opposite direction, i.e. infection rate reflects mosquito abundance? 

Is the work clearly and accurately presented and does it cite the current literature?

Partly

If applicable, is the statistical analysis and its interpretation appropriate?

Partly

Are all the source data underlying the results available to ensure full reproducibility?

Yes

Is the study design appropriate and is the work technically sound?

Yes

Are the conclusions drawn adequately supported by the results?

Yes

Are sufficient details of methods and analysis provided to allow replication by others?

Yes

Reviewer Expertise:

Biological timing of mosquito vectors

I confirm that I have read this submission and believe that I have an appropriate level of expertise to confirm that it is of an acceptable scientific standard, however I have significant reservations, as outlined above.

## References

[1] : Investigating associations between biting time in the malaria vector Anopheles arabiensis Patton and single nucleotide polymorphisms in circadian clock genes: support for sub-structure among An. arabiensis in the Kilombero valley of Tanzania. *Parasit Vectors*.2016;9: 10.1186/s13071-016-1394-8 109 10.1186/s13071-016-1394-8 26920563PMC4769569

[2] : Frequent blood feeding enables insecticide-treated nets to reduce transmission by mosquitoes that bite predominately outdoors. *Malar J*.2016;15: 10.1186/s12936-016-1195-8 156 10.1186/s12936-016-1195-8 26969430PMC4788858

[3] : Determinants of host feeding success by Anopheles farauti. *Malar J*.2016;15: 10.1186/s12936-016-1168-y 152 10.1186/s12936-016-1168-y 26964528PMC4785651

[4] : Anopheles farauti is a homogeneous population that blood feeds early and outdoors in the Solomon Islands. *Malar J*.2016;15: 10.1186/s12936-016-1194-9 151 10.1186/s12936-016-1194-9 26960327PMC4784415

[5] : Successful malaria elimination strategies require interventions that target changing vector behaviours. *Malar J*.2013;12: 10.1186/1475-2875-12-56 56 10.1186/1475-2875-12-56 23388506PMC3570334

# Response

**Published:** 2019-10-29

**Authors:** irfanul chakim

**Affiliations:** Chulalongkorn University, Indonesia

**Comments by reviewer of the F1000 research:**

***The diversity of Anopheles blood feeding patterns suggests different malaria protection strategies in different localities [Version 1; peer review: Approved with reservations]***

Author:

We thank to the reviewer for his positive feedback. We do agree with these comments.

Major comments

No: 1

Comments: We did not use ANOVA as suggested by reviewer due to our data was not normal based on normality test. Therefore, to run a multiple comparison test based on not normal data set, we need to do it by using a non-parametric test. Hence, Kruskall-wallis is a non-parametric test for multiple comparison test and Dunn’s is a non-parametric post hoc test.

No: 2

Action: We deleted such reference “cicardian clock genes” from the paragraph as suggested by reviewer.

No: 3

Action: References have been added to the text in the discussion section.

No: 4

Action: The information has been added in the discussion section.

Minor comments

No: 1

Action: Revision has been made according to reviewer suggestion.

No: 2

Action: We have changed the word “mosquitos” to “mosquitoes” throughout text accordingly.

No: 3

Action: We have italicized the word “Anopheles” throughout text accordingly.

No: 4

Action: Information has been added to the text accordingly.

No: 5

Comment: These are SD bars on the charts

No: 6

Comment:We agreed with the reviewer.

Action: Therefore we changed the sentence to “The difference in the annual incidence rate of malaria infection may also reflect mosquito abundance in different endemic areas.”
